# Metabolomic Studies for the Evaluation of Toxicity Induced by Environmental Toxicants on Model Organisms

**DOI:** 10.3390/metabo11080485

**Published:** 2021-07-27

**Authors:** Hyung Min Kim, Jong Seong Kang

**Affiliations:** College of Pharmacy, Chungnam National University, Daejeon 34134, Korea

**Keywords:** metabolomics, environmental pollutant, model organisms, phenotype assay, toxicity

## Abstract

Environmental pollution causes significant toxicity to ecosystems. Thus, acquiring a deeper understanding of the concentration of environmental pollutants in ecosystems and, clarifying their potential toxicities is of great significance. Environmental metabolomics is a powerful technique in investigating the effects of pollutants on living organisms in the environment. In this review, we cover the different aspects of the environmental metabolomics approach, which allows the acquisition of reliable data. A step-by-step procedure from sample preparation to data interpretation is also discussed. Additionally, other factors, including model organisms and various types of emerging environmental toxicants are discussed. Moreover, we cover the considerations for successful environmental metabolomics as well as the identification of toxic effects based on data interpretation in combination with phenotype assays. Finally, the effects induced by various types of environmental toxicants in model organisms based on the application of environmental metabolomics are also discussed.

## 1. Introduction

With the development of modern technology, mass production has become common, resulting in the generation of large quantities of waste. For example, globally, approximately 242 million tons of plastic wastes are generated annually [1]. Thus, environmental pollution, which is becoming a serious problem, continues to worsen. Many researchers have shown interest in the risk assessment of environmental pollutants for decades and studied the toxicity of these pollutants. In this regard, various types of model organisms living in polluted environments have been exposed to target toxicants to study the effects of these toxicants in these model organisms. Furthermore, representative environmental toxicants including pharmaceuticals and personal care products (PPCPs) and pesticides, have been observed in aquatic environments such as seas and rivers [2,3,4,5,6]. The toxicity of these compounds to marine organisms has also been extensively studied. For example, in previous studies, *Oryzias melastigma*, a widely used marine fish model, was exposed to various types of pollutants, including bisphenol A (BPA), benzo[a]pyrene, and nanoparticles. These can lead to toxic effects that inhibit reproductions, induce metabolic disorders, and cause inflammation [7,8,9,10]. Additionally, toxicity in several other marine organisms, such as copepods, clams, and microalgae, has been widely investigated [11,12,13].

Environmental pollution appears not only in the ocean, but also in soil and air [14,15]. Representative model organisms, including earthworms and *Caenorhabditis elegans* (*C. elegans*), have been used to investigate the toxicity of different soil pollutants on soil organisms. These soil organisms were exposed to pollutants [16,17,18,19], and researchers observed consequences on the growth, reproduction, and various metabolic pathways in these organisms. The toxicity of fine dust, which is a representative pollutant in air, as well as those of other air pollutants has been extensively studied. For example, a rat model was exposed to air pollutants, and the induction of stress in the overall pathways related to life maintenance was observed [20,21]. Many environmental toxicity studies have been conducted with a primary focus on phenotypes and specific biochemical processes (Table 1). Such biochemical studies often involved the investigation of behaviors assays, including locomotion, swimming, physiological, and avoidance behaviors, and biochemical responses, such as histology, transcriptomes, and oxidative stress. Furthermore, in phenotype-based studies, investigations can be conducted using a limited range of designated targets. However, such an approach can lead to the acquisition of limited information compared to the use of a more comprehensive approach.

Metabolomics is defined as the comprehensive analysis of small molecule metabolites (<1000 Da) in various organisms [22,23]. It involves the evaluation of the perturbation of metabolic reactions in various experimental models, such as the evaluation of disease states and exposure to xenobiotics, including pharmaceuticals, and many other toxic compounds. The key biochemical responses of the experimental model can then be determined by identifying the biomarker metabolites. Environmental metabolomics is the study of how environmental conditions affect the metabolic processes in an organism [24,25]. By analyzing metabolite levels in model organisms in environments where the pollutants are primarily distributed, it is possible to comprehensively evaluate the actual impact of pollutants on the environment. This approach can enhance our understanding of the effects of environmental pollutants on the physiological changes that occur in living organisms, as well as the associated response mechanisms [26,27]. In this regard, public interest in the environment has increased, and environmental metabolomic studies are receiving increasing attention. According to a previous report, there has been a steady increase in the number of published environmental metabolomics-related studies since 2000. Approximately 900 papers on this topic are published annually [28]. In this review, we provide an overall information on environmental metabolomics. This includes the workflow of metabolomics, discussion on the types of model organisms and environmental pollutants used for toxicity studies, and suggestions for future research directions for environmental metabolomics.

**Table 1:** Recent representative toxicity study in biochemical processes.

Year	Model Organisms	Toxicants	Biochemical Study	Main Findings	References
2017	*C. elegans*	Rare earth elements	Neurotoxicity	Rare earth elements are now widely used in daily life. Trichloride neodymium, praseodymium, and scandium induced loss of dendrite in dopaminergic and GABAergic neurons and downregulated *dat-1::GFP* and *unc-47::GFP i*n *C. elegans*.	Xu et al. [29]
2017	*D. rerio*	Heavy metals	Swimming and AChE activity	Exposure of cadmium toward zebrafish strongly inhibited acetylcholinesterase (AChE) activity in the gill of zebrafish and decreased swimming behavior, which might be an evidence of neurotoxicity.	Pan et al. [30]
2017	*D. rerio*	Fine particulate matter	Multi-organ toxicity	This study evaluated toxicity of fine particulate matter (PM2.5) in a zebrafish. PM2.5 induced embryonic toxicity, hepatotoxicity, and neurotoxicity on model organisms.	Duan et al. [31]
2018	*C. elegans*	Phthalates	Multigenerational toxicity	Phthalates induced multigenerational toxicity regarding locomotive effects and total brood size, which might be related to disruption of vitellogenin and H3Kme2 demethylase.	Li et al. [32]
2018	*E. fetida*	Insecticides	Avoidance behavior and reproduction	Toxic effect of insecticides toward earthworms were studied. As a result, avoidance behavior was observed along with decrease of reproduction.	Ge et al. [33]
2018	*L. fortunei*	Herbicides	Biochemical responses	This study evaluated biochemical responses of the golden mussel *Limnoperna fortunei* upon exposure to glyphosate. Dietary exposure of glyphosate altered detoxification responses; however, it did not affect oxidative stress parameters.	Iummato et al. [34]
2019	*D. rerio*	Pharmaceuticals	Embryonic development and biochemical effects	Effects of environmental relevant levels of Paracetamol and Ciprofloxacin on zebrafish were evaluated. These pharmaceuticals affected developmental process, behaviors, epigenetics, and enzyme activities.	Nogueira et al. [35]
2020	*C. elegans*	Nanopolystyrene	Locomotion and sensory systems	Nanopolystyrene induced toxic effect on locomotion and sensory systems, specifically on development of dopaminergic neurons.	Wang et al. [36]
2020	*D. rerio*	Personal Care	Transcriptional, biochemical, and histological effects	Biochemical Effects of Benzotriazole UV stabilizer on zebrafish were evaluated. The compound altered level of antioxidant enzymes, expression of stress response gene, and induced damage on liver.	Hemalatha et al. [37]
2020	*D. magna*	Organometallic biocide	Biochemical Effects	Toxicity of Zinc pyrithione (ZnPT) in *Daphnia manga* was evaluated regarding biochemical effects. As a result, ZnPT induced oxidative and neurotoxic effects, which may be a potential threat to aquatic organisms.	Sousa et al. [38]
2020	*D. magna*	Drink water treatment residue	Physiological and biochemical responses	Drink water treatment residue (DWTR) is a byproduct produced during drinking water production. Adverse effects induced by DWTR was evaluated. The study evaluated effects of DWTR on the survival, growth, reproduction, body morphology, and oxidative stress.	Yuan et al. [39]
2020	*E. eugeniae*	Pesticides	Physiological behavior	Exposure of pesticides on earthworms induced decrease in reproductive activity, rupture of muscles and tissues, and increase in mortality rate.	Gowri et al. [40]
2021	*A. parthenogenetica*	PAHs	Lethal, behavioral, growth and developmental toxicities	Polycyclic aromatic hydrocarbons (PAHs) are one of widespread pollutants in aquatic environments. The effects of these compounds toward brine shrimp were evaluated. Survival, behavior, and growth were affected upon exposure to PAHs and body length could be used as an indicator for the evaluation of development.	Cong et al. [41]
2021	*C. pyrenoidosa*	Nanoplastics	Growth, photosynthesis, and oxidative stress	Toxic mechanisms of nanoplastics on microalgae (*Chlorella pyrenoidosa*) was investigated. By transcriptomic analysis, nanoplastics could be responsible for decreased gene expression of aminoacyl-tRNA synthetase. Algae have detoxification mechanisms by regulating intracellular osmotic pressure.	Yang et al. [42]

## 2. Application of Metabolomics in the Study of Biological Responses to Environmental Toxicants

Metabolomics is the comprehensive study of metabolite levels in model organisms or target organs based on different experimental designs. Biochemical perspective results can be obtained by analyzing the levels of small molecule metabolites. Additionally, the effects of environmental stressors on model organisms can be evaluated to determine the relationship between phenotypes and altered metabolic pathways. To achieve a better understanding, metabolomic studies can be combined with other omics studies, including genomics, transcriptomics, proteomics, and epigenomics. With multi-omics approaches, the association between phenotypes and metabolites can be confirmed. Moreover, the integration of metabolic pathways and protein, gene, and transcript data can be useful in elucidating the mechanism of action of a given toxicant in a specified experimental model.

Various analytical techniques have been employed for metabolomics studies. For example, mass spectrometry (MS) and nuclear magnetic resonance (NMR) spectroscopy are the most widely used analytical methods in metabolomic studies [43,44]. With technological advances, the performance of these instruments, with respect to precision and sensitivity, has been improved. Thus, there has been an increase in the quality of information obtained via instrumental analysis. NMR has been widely used owing to its high reproducibility and non-destructive nature [45,46]. However, this technique shows a relatively low sensitivity; hence, it requires samples with concentrations in the millimolar range for the collected data to be of significance. Several analytical approaches for the application of NMR have also been reported. Specifically, the two-dimensional approach offers the possibility to improve sensitivity, and is advantageous as it provides additional information about the samples [47]. Furthermore, high-resolution magic angle spinning (HRMAS) NMR, which has been employed in several metabolomics-related studies, has enabled the analysis of liquid and solid samples [48,49].

The most widely used instrument in metabolomic studies is MS, which is suitable for high-sensitivity and high-throughput analysis, and it can be applied in various research fields involving different types of samples [50,51]. MS offers the possibility of analyzing all ionizable molecules in a sample based on the mass-to-charge ratio. It also allows the realization of a relative or absolute quantitative comparison between samples [52]. However, unlike NMR, MS has the disadvantage of being a destructive technique. Furthermore, given that metabolites are chemically diverse, they can be analyzed using liquid chromatography (LC), gas chromatography (GC), or capillary electrophoresis (CE) equipped with MS based on their properties [53,54,55]. For example, in LC, non-volatile compounds can be separated based on their polarities, and in GC, the separation of metabolites is based on their boiling points, while in CE, the separation is based on the mobility of the generated ions [56,57]. LC-MS and GC-MS have been frequently used, and when this is the case, each instrument can be used to analyze various types of metabolites. On the one hand, GC-MS has advantages such as good separation, reproducibility of retention time, and ease of operation. In particular, GC-MS primarily uses the electron ionization (EI) method, which is a hard ionization process, and the obtained MS spectrum has excellent reproducibility for each compound. The characteristic of producing reproducible MS spectrum of GC-MS has led to well-established libraries of thousands of compounds [58]. Therefore, unknown compounds can be identified by matching this spectrum with libraries. This feature makes the identification of unknown compounds in GC-MS more straightforward than the LC-MS approach. Nonetheless, GC-MS can only be used to analyze volatile compounds; thus, a derivatization step is often necessary during the analysis of key metabolites, such as amino acids, sugars, and fatty acids [59,60].

On the other hand, LC is a high-sensitivity and high-selectivity analysis method suitable for the analysis of non-volatile substances [61,62]. The most frequently used ionization technique in LC is the electrospray ionization (ESI) method, which offers the possibility to analyze macromolecules, given that it can produce multiple charged ions. However, it is unsuitable for the analysis of nonpolar compounds. Unlike the EI method, the ESI method is a soft ionization technique. Hence, it is easier to determine the molecular mass of the parent ion [63,64]. In addition, the coupling of MS with LC is possible with both low- and high-resolution mass spectrometers, for which resolution, expressed as R = m/Δm, is defined as the smallest difference in mass that can be separated based on the *m*/*z* of interest [65]. Low-resolution MS (LRMS) provides a resolution of approximately 1000, while a high-resolution mass (HRMS) has a resolution of over 5000 and may show performance above 100,000, depending on the type of instrument used [22,66]. LRMS offers the possibility to distinguish *m*/*z* down to the atomic mass units (amu). Moreover, the representative MS analyzer for LRMS is a triple quadrupole and ion trap, which is primarily used for the realization of targeted metabolomics and offers the possibility to realize an absolute quantitative approach based on the selective analysis of major metabolites using authentic standards. Furthermore, it enables a relative comparison using a semi-targeted approach based on the application of hundreds of multiple reaction monitoring (MRM) transitions as well as advanced data processing [67]. Representative HRMS analyzers include time-of-flight (ToF), orbitrap, Fourier-transform ion cyclotron resonance (FT-ICR), and hybridized tandem MS coupled with quadrupole, and ion traps, including Q-ToF, and Q-Orbitrap [68,69,70]. HRMS offers the possibility to measure *m*/*z* values down to the fourth decimal place; hence, it shows superior identification performance. Therefore, an untargeted approach is most frequently applied with HRMS, which performs identification via data processing after analysis without the specification of analysis targets [71,72]. Such an untargeted method involves complex data processing and requires significant time and effort for the identification of metabolites. Nevertheless, it is the most widely used analysis method because it allows the comparison and analysis of hundreds of metabolites in an unbiased manner.

## 3. Metabolomics Workflow

**Figure 1:**
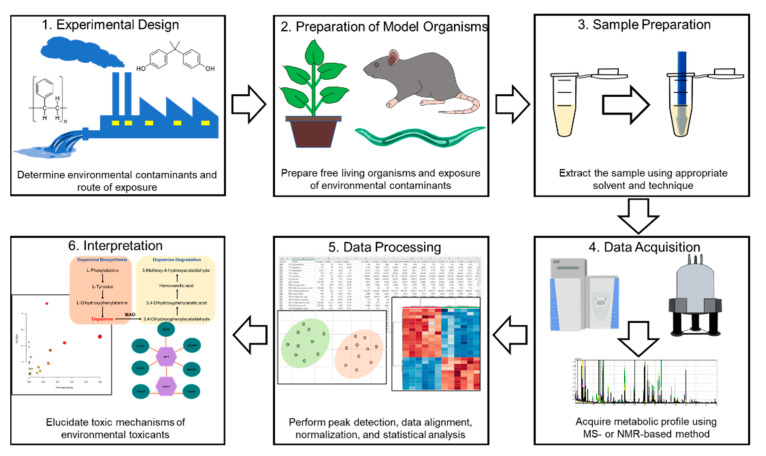
Representative workflow of environmental metabolomics.

A typical metabolomics workflow scheme is shown in Figure 1 from which it is evident that the first step is to build an experimental model. In this regard, it is necessary to select an appropriate external stressor for the target metabolite. In addition, suitable model organisms that show a correlation with the characteristics of external stressors must be selected. Thereafter, the appropriate sample is collected, followed by optimization of the extraction of the metabolites from the collected samples [73]. Extracted metabolites can be separated using an instrumental separation technique (LC, GC, etc.), and data will be acquired via high-throughput detection methods (MS, NMR, etc.). Thereafter, by applying sophisticated bioinformatics tools, the lists of significantly affected metabolites can be generated from a complex list of data, and based on the interpretation of the results, it then becomes possible to gain a comprehensive understanding of the effects of external stressors in the model organism. In the experimental process, optimization of extraction method, analytical conditions, and data processing methods and parameters significantly influence the quality of the results. Therefore, to achieve the best results, it is necessary to work with optimal methods based on an understanding of the entire workflow.

### 3.1. Preparation of Model Organisms

To achieve a valuable research output, it is important to establish optimal experimental models. Specifically, in environmental metabolomics, the first important factor to consider is the selection of an external stressor [74]. External stressors should be selected according to the goals of the study. It is also important to identify the ecosystem in which the corresponding external stressors are mainly distributed. Additionally, setting up a model with relevant concentrations and studying major organs that are related to the associated toxic pathways is crucial. It is also important to select a proper target organ, given that toxicant accumulation and exposure routes can differ, depending on the chemical properties of external stressors. There are two different methods by which model organisms can be established: (i) collecting organisms directly from the environment for the analysis, and (ii) constructing an experimental model in a laboratory environment. If the goal is to perform research on actual conditions, the best strategy is to collect organisms directly from the environment. However, the disadvantage of such an approach is that it could be associated with other variables, given that the culturing conditions of the organism cannot be controlled. Hence, changes in metabolite levels could be caused not only by external stressors, but also by various environmental variables. Conversely, when experiments are conducted in a controlled laboratory environment, all working conditions can be kept constant, except for external stressors. Thus, it is possible to analyze changes in the concentrations of metabolites that are solely affected by external stressors. Therefore, it is crucial to establish an appropriate experimental model based on the study objectives.

### 3.2. Sample Treatment

The extraction of metabolites from samples is a critical step in environmental metabolomic studies. During this step, it is important to extract a large number of metabolites from biological samples using an optimized extraction process. This is because the application of a proper and reproducible extraction method can lead to an accurate interpretation of the obtained results. Furthermore, extraction methods can be optimized based on the characteristics of the target metabolites as well as those of the analytical methods. To extract metabolites, it is necessary to disrupt the samples in a solvent using a homogenizer by considering the target tissue or the model organisms [75,76]. It is also necessary to identify an optimal homogenizer via comparative analysis, because the homogenization efficacy differs based on the type of sample under consideration. For example, to extract metabolites from *C. elegans*, the efficacy of the extraction processes corresponding to six types of homogenizers and two types of solvent compositions were compared, and extraction with 80% methanol and a methanol/chloroform mixture yielded reproducible results. Homogenization with a bead beater showed the highest total number of metabolites, precision, and yield, as well as high throughput with minimal variation during sample preparation steps [77]. Additionally, it is essential to carefully determine the composition of the extraction solvent. In a recent study, extraction methods that yield hydrophilic and lipophilic layers have been applied, and a simultaneous extraction procedure for the profiling of polar and mid-polar metabolites has also been reported. Medina et al. recommended that a mixture of isopropanol and 1-butanol:methanol worked best for the analysis of lipids and polar metabolites from human plasma [78,79]. Therefore, it is imperative to apply a customized extraction method that considers the polarity of the metabolites of interest. Owing to the difference in ionization and separation methods, some compounds are difficult to detect with high sensitivity in LC-MS [80]. In such a case, analysis can be performed using GC-MS with chemical derivatization, which is a step that is selected based on the functional groups present in the target metabolites [81]. After the extraction of metabolites, further sample clean-up steps, such as solid phase extraction (SPE), can be employed [82,83]. Unfortunately, these sample optimization steps are unsuitable when an untargeted approach is used, because they can lead to the acquisition of biased results. However, when there is a clear target metabolite, performing these steps is recommended because they can concentrate the sample, which can further lead to high sensitivity owing to sample enrichment. Moreover, these sample work-up processes require effort to minimize sample variability via the application of a consistent method and time for their analysis.

### 3.3. High-Throughput Techniques for Metabolite Screening

Metabolomic studies can be performed using high-throughput techniques, such as MS and NMR, which are currently the most widely used techniques in this regard. With such high-throughput approaches, information can be obtained from thousands of metabolites. Because these metabolites have different chemical properties, it is often necessary to apply several analytical methods to obtain comprehensive results. In addition, during the extraction of metabolites from biological samples, thousands of metabolites may be present in a single extract. Therefore, when using an untargeted metabolomics approach, it is often necessary to separate the metabolites as much as possible, based on their chemical properties using an analytical column. Prior to MS detection, LC and GC are used to achieve efficient separation of the metabolites. Thereafter, thousands of metabolites present in the sample are eluted based on an order that is related to their chemical properties in the column. These metabolites are transferred to MS to generate a mass spectrum. LC columns can be classified based on column chemistry as a reverse phase or hydrophilic interaction liquid chromatography (HILIC) column (Table 2) [84,85]. Specifically, the reverse phase column, which is the traditional design, consists of a nonpolar stationary phase with a polar mobile phase, for example, in C18, which is the most widely used stationary column, water, methanol, and acetonitrile are the most frequently used mobile phases [86]. Therefore, nonpolar to mid-polar metabolites are retained in the stationary phase, while polar substances are eluted first. Additionally, in metabolomics, some of the important metabolites, including amino acids and carbohydrates, which are polar and hardly retained in reverse phase columns, are eluted simultaneously near the dead volume. To make improvements in this regard, a new strategy that involves different compositions of the stationary phase, known as HILIC, has emerged. The HILIC column enables the analysis of polar metabolites, which are difficult to detect using the traditional C18 column; hence, it can be applied in a complementary manner [87,88]. Thus, a broader spectrum of metabolites can be identified when the results of both methods are combined. In addition to the HILIC approach, GC-MS analysis with derivatization has long been the preferred method for the analysis of polar primary metabolites. Amino acids and carbohydrates have also been analyzed using various silylation agents, when methylation agents are often preferred for the analysis of fatty acids [59,81,89]. Furthermore, metabolites that can be analyzed via GC-MS are often involved in key metabolic pathways related to energy production, such as glycolysis, TCA cycle, and free fatty acid metabolism, in model organisms [90,91]. 

Based on performance, MS can be classified as LRMS or HRMS, and the approach employed in metabolomic studies depends on the type of mass spectrometer used [92,93]. Specifically, when using untargeted metabolomics approaches, the identification of unknown compounds using *m*/*z* ratios based on LRMS techniques, such as QqQ MS, is challenging, because LRMS can only accurately measure integer values. Conversely, HRMS techniques, such as QToF and Orbitrap, can accurately perform measurements down to four decimal places. Thus, they offer the possibility of identifying unknown compounds based on their *m*/*z* values at a higher resolution. Furthermore, when working with LRMS techniques, such as QqQ, targeted metabolomics with absolute quantification or semi-targeted metabolomics can be applied [94,95,96]. The targeted metabolomics approach has the advantage of enabling the evaluation of the effects of different metabolites on specific metabolic pathways by targeting a few selective metabolites. For the semi-targeted approach, a QqQ mass spectrometer scans and compares hundreds of metabolites within a short time using the multiple reaction monitoring (MRM) method, mainly targeting hundreds of known metabolites. This is neither an absolute nor an untargeted method. However, it is a powerful approach that allows a comparative analysis of a large number of metabolites using LRMS [67,97]. HRMS has the advantage of high resolution; thus, it can detect *m*/*z* values in decimal digits, which is advantageous for the identification of unknown compounds. Additionally, a comprehensive analysis of metabolite extracts can be achieved via data-dependent analysis (DDA) or data-independent analysis (DIA), which can yield hundreds to thousands of peaks [98,99]. Thus, the identification process can be easily processed using highly advanced data processing techniques and by comparing established online or in-house libraries [100,101]. Furthermore, with the advances in analysis and data processing techniques, it is now possible to obtain much higher quality data than those obtained in the past.

Additional considerations are needed to achieve reliable high-throughput screening. Quality assurance (QA) and quality control (QC) are essential processes for achieving satisfactory metabolomics research results [23,102]. QA is often performed to ensure that metabolomic studies have been conducted, considering the essential elements that are needed to obtain qualified data. In contrast, QC requires that every experimental step is performed to ensure the acquisition of quality data. There is a representative guideline for QA/QC, known as “Guidance for Industry: Bioanalytical Method Validation” published by the Food and Drug Administration. Given that this guideline was established for the purpose of target drug analysis, it can be applied in either targeted or semi-targeted metabolomics. Several research groups are currently working to standardize the QA/QC processes for metabolomic studies [103,104]. With the harmonization of QA/QC processes, the reproducibility of metabolomics can be improved, leading to an increase in the reliability of the acquired results. Therefore, it is necessary to consider these processes for acquiring high-quality environmental metabolomics data.

### 3.4. Data Processing

When performing metabolomic studies using an untargeted approach, applying optimized data processing techniques is necessary to ensure satisfactory results. Such data processing is performed by employing procedures such as feature detection, background correction, noise reduction, alignment, and normalization [105,106,107]. Complementary online software, such as MZmine, MS-DIAL, and XCMS, have been applied for the aforementioned procedures [108,109,110]. Additionally, proprietary software from vendors, including Compound Discoverer, Progenesis QI, and MassHunter, are available. These data processing tools provide peak information and intensity values, which can be employed via univariate and multivariate analyses to identify statistically significant metabolites. Principal component analysis (PCA), which is an unsupervised method, can provide information on the overall clustering behaviors of the metabolic profiles of different samples [111]. It can also provide unbiased data that can be used when evaluating general trends corresponding to the control and treated data. Partial least-squares discriminant analysis (PLS-DA) is a supervised method that is widely used for feature selection and classification [112]. In general, these multivariate tools have been extensively used in omics studies to identify statistically significant metabolites. Besides the above-mentioned multivariate approaches, univariate analyses are performed for biomarker identification, among which the *t*-test and ANOVA are the most frequently employed methods [113]. Statistically significant metabolites have also been identified by comparing with mass spectra databases, including mzcloud, Metlin [114], lipidmaps [115], and hmdb [116] or by comparing with an in-house library established through the analysis of authentic standards. Such an identification process can also be performed by comparing accurate mass and MS/MS fragmentation results with existing databases.

### 3.5. Interpretation of Data

Metabolites can be identified through data processing, and further analysis can lead to the generation of a list of significant metabolites. With these data, interpretation can be performed by further analysis, using methods such as pathway analyses [117]. This will help to identify the metabolic pathways that include significantly altered metabolites and elucidate the potential toxic mechanisms. Given that metabolic pathways are intricately linked, the perturbation of a single metabolite can affect a series of biological processes, and perturbated metabolic pathways can be clarified by interpreting omics data. Thus, the comprehensive toxic effects of environmental pollutants could be determined. 

In addition to metabolomics results, conventional phenotype studies can be of great help in interpreting the results of metabolomic studies. By combining metabolite identification results with those of phenotype studies, it is possible to confirm changes in metabolite levels and report various phenotypes based on the observation of the model organisms employed. Phenotypic studies have been conducted over long periods to identify the effects of exposure to toxic substances [118,119,120]. However, the association between changes in phenotypes and biochemical processes can only be elucidated by conducting in-depth studies. Furthermore, metabolomic studies can enable the identification of metabolic pathway perturbations. When these results are translated in combination with phenotype analysis, the toxic effects of environmental pollutants in model organisms can be clarified more comprehensively. For example, in a study conducted by Kim et al., exposure of *C. elegans* to nanopolystyrene resulted in phenotypic changes, such as oxidative stress, reproduction, and locomotion [18]. A metabolomics study has been performed to identify perturbations in energy-related metabolism. Accordingly, the association between a decrease in the reduction rate and energy metabolism was confirmed. In addition to metabolomic studies, in-depth studies on biochemical pathways can be achieved using multi-omics approaches, such as lipidomics, transcriptomics, proteomics, and epigenomics. Overall, the combination of these approaches can facilitate a comprehensive understanding of the toxic effects of environmental pollutants and provide clues regarding the future direction of related research.

## 4. Exposure on Emerging Environmental Pollutant

Environmental pollutants, also known as xenobiotics, are chemical substances found in living organisms, and are not naturally produced by organisms [121,122]. There are several different types of xenobiotics, including PPCPs, pesticides, and plastics [123,124]. These substances can be ejected from factories, homes, and hospitals and released into the environment; released via the excrement of organisms’ derived from landfills; or drained from agricultural water and factory wastewater [125]. These xenobiotics, which are widely used in daily life, cannot be decomposed in poorly functioning wastewater treatment plants, and because of their stable nature, their degradation is often very slow [126,127]. Furthermore, xenobiotics can exist in the environment for a long period and continue to circulate. They can also exist in discharged forms and undergo various transformation processes via biological and chemical degradation [128,129]. Thus, substances with different chemical properties are often observed in various environments as pollutants. This implies that it is important to elucidate how such substances affect organisms, based on their route of exposure. In this section, existing model organisms and the current situation regarding emerging contaminants are discussed. Based on studies conducted to confirm their toxicity of those contaminants, the reported toxic mechanisms are also summarized (Table 3).

### 4.1. Model Organisms for Environmental Metabolomics

Choosing an appropriate model organism is critical to obtain reliable results in toxicity studies. Environmental pollutants are mostly found in water-based environments, such as wastewater and drinking water; however, they can also flow into the soil via irrigation or several other means [130]. Additionally, pollutants, such as pesticides, have been detected in soils at high concentrations, given that they are predominantly applied in soil-based environments. To evaluate the environmental impact of pollutants in oceans, the effects of exposure of marine organisms to pollutants have been examined. In this regard, *Danio rerio*, also known as zebrafish, is widely used as an aquatic model organism in toxicological studies [131]. This model has several advantages, including cost-effective breeding conditions, small space occupation, similarity of genome with humans, and high productivity. It also exhibits well-conserved developmental and physiological processes, similar to those observed in humans. Thus, the similarities between the genes and phenotypes of this model and those of humans make it a good model organism for disease and toxicity studies [132,133]. Another famous model is *Daphnia magna*, which is one of the oldest model organisms used in biological research [134]. The toxicity of this model can be evaluated based on various types of behavioral and physiological responses [135,136]. Moreover, their phenotypes can be used as biomarkers to evaluate the toxic effects of environmental pollutants. These factors make *D. magna* a promising model organism for environmental toxicology studies. To evaluate the effect of toxicants on the soil environment, toxicity resulting from the exposure of soil organisms, such as earthworms, *C. elegans*, or plants, on pollutants has been examined. Specifically, *C. elegans*, a free-living nematode found in soil systems, has been widely used for toxicity studies because of its transparent body, well-characterized genome, ease of maintenance, and sensitivity to toxicants [137,138]. Additionally, this soil organism shows various phenotypic characteristics, including development, reproduction, locomotion, oxidative stress, cell apoptosis, and stress responses, which can be assessed by applying mutant strains [138,139]. Therefore, *C. elegans* has been widely used to evaluate the toxic effects of various types of environmental toxicants, such as pharmaceuticals, pesticides, and nanomaterials. When environmentally relevant organisms are used, the results of such studies can be applied to improve environmental health. The output of these studies can impact society, and their application can lead to policy changes, a decrease in the usage of pollutants, and a deeper understanding of the underlying mechanisms governing toxicant action.

### 4.2. Pharmaceuticals and Personal Care Products

PPCPs are used to prevent or treat diseases in humans and animals, as well as to improve the quality of life. Owing to their widespread use every day, a large number of compounds have been detected as pollutants in various environmental media, including wastewater, drinking water, sludge, and soil [140,141,142,143,144,145]. As these pollutants are detected in various environmental conditions, they cause various hazards. Recently, studies have been conducted on their toxic effects. In particular, the ocean is one of the places where environmental pollution is severe. Several metabolomic studies involving marine organisms exposed to pharmaceuticals have been conducted. One study focused on the toxicity of antibiotics on the embryos and larvae of *D. rerio* [146]. The metabolites in these organisms were analyzed using LC-QToF-MS, and the results indicated that some key metabolites were regulated upon exposure to antibiotics. These are representative studies that highlight the effects of pharmaceuticals on the behavior and metabolism of marine organisms using metabolomics and phenotype assays. In another study, the impact of PPCPs on biological pathways in zebrafish embryos was evaluated by combining metabolomics with gene expression studies [147]. The obtained results clearly elucidated the perturbation of biological pathways due to exposure to PPCPs, which reportedly also exerted effects on nitrogen metabolism, energy metabolism, fatty acid synthesis, and phenylalanine, tyrosine, and tryptophan biosynthesis. Additionally, exposure to these compounds can dysregulate the central nervous system. This could be a serious problem for the organisms exposed in the environment.

When waterways are contaminated and the contaminated water flows into the soil, the living species present in the soil can be affected. Thus, metabolomic studies have been conducted to clarify the effects of toxicants on soil organisms such as earthworms and *C. elegans*. Triclosan is one of the most widely used PPCPs. It is classified as an antibiotic and frequently used in personal care and household products to eliminate bacteria [148]. Furthermore, its effect on soil organisms, including earthworms and *C. elegans*, has been evaluated using GC-MS based metabolomics [149]. It has been observed that upon exposure to triclosan, earthworms lose weight and show increased mortality. Additionally, exposure to triclosan resulted in a significant alteration in the amino acid and polyamine levels in earthworms. In *C. elegans*, exposure to triclosan induces a decrease in lifespan, reproduction, and locomotion. An increase in oxidative stress has also been reported, and metabolic pathways, including tyrosine metabolisms and alanine, aspartate, and glutamate metabolisms were significantly affected [150]. Therefore, the perturbation caused by metabolites owing PPCP exposure is of great significance, given that these compounds are frequently detected as environmental contaminants. This implies that studying the harmful effects induced by these compounds can facilitate the estimation of the effects of the detected pollutants. Therefore, metabolomic studies can deepen the understanding of the toxic effects of PPCPs in various environments that are of interest to the public.

### 4.3. Pesticides

The primary role of pesticides is to kill, interfere, and reduce the spread of pests, such as insects and rodents [151]. According to previous studies, approximately 2 million tons of pesticides are consumed annually, and since most of these pesticides are used for agricultural purposes, they often exist in the soil at high concentrations [152]. Although the use of pesticides with severe toxicity has been prohibited, widely used pesticides can still exert toxic effects in unspecified organisms. Therefore, the influence of these pesticides on soil organisms has been confirmed in several studies. For example, LC–MS-based metabolomics was performed to evaluate the effects of sulfoxaflor in earthworms [153]. The results of this study showed that exposure to sulfoxaflor induced oxidative stress in earthworms. This was confirmed by measuring the activities of antioxidant enzymes and malondialdehyde accumulation. Furthermore, using metabolomics, the perturbation of energy metabolism, urea cycle, and nucleotide metabolism in earthworms by pesticides has been confirmed. Therefore, given that earthworms play an important role in soil systems, further evaluation of the risk of pesticides in the environment is necessary.

The impact of pesticides has also been studied in plant species that grow in soil environments [154,155]. The presence of pesticides in the soil can lead to the accumulation of pesticides in crops, which could affect human health and threaten food safety. Therefore, it is necessary to assess the accumulation and potential toxic effects of pesticides in crops. In a recent study, the impact of three representative pesticides, butachlor, chlorpyrifos, and tricyclazole, on *Oryza sativa L.* were evaluated using metabolomics and transcriptomics approaches [156], and the perturbation of carbohydrate, amino acid, and fatty acid metabolism was confirmed. The results of such studies can facilitate the evaluation of environmental risk assessment efforts and emphasize the need to control pesticide usage.

### 4.4. Nanoparticles

Nanoparticles are grouped into two classes, namely, engineered nanoparticles, which are made for specific purposes, and secondary nanoparticles, which are produced by the abrasion of plastic contaminants [157]. With advances in technology, engineered nanoparticles are used in various industries, including the medical, health, and electronics fields. Therefore, there has been an increase in nanoparticle-related environmental pollution. Given their widespread use, more than 300 million tons of plastics are produced annually, with 2–5% of the resulting plastic waste ending up in the soil and ocean [158,159]. In particular, nanoparticles exhibit various physical properties based on their compositions, and they could possibly adversely affect unspecified organisms, depending on their properties [160,161]. Therefore, it is necessary to investigate the effects of the frequent use of nanoparticles. Furthermore, given that nanoparticles have unique physicochemical properties depending on their composition, it is important to elucidate their effects on the environment and in model organisms. In this regard, metabolomics can be employed to clarify the metabolic changes in model organisms from different environments (aquatic, soil, etc.). Thus, the potential toxicity of nanoparticles toward different environmental media, as well as the possible related mechanisms of action in living organisms can be elucidated.

The perturbation of metabolite levels in mussels following exposure to nanoplastics has been evaluated [162]. Specifically, metabolomic studies have revealed the disruption of phenylalanine metabolism in mussels, which could induce oxidative stress and neurotoxicity. Additionally, biochemical studies have confirmed the oxidative stress and immunotoxicity caused by exposure to nanoplastics. In another study, the effects of 10 types of nanoparticles on rice were evaluated to clarify the environmental risks using an untargeted approach [163]. The results showed that carbohydrate metabolism was downregulated, while amino acid metabolism was upregulated. Moreover, network analysis revealed a relationship between the characteristics of nanoparticles and perturbation of metabolic pathways. Furthermore, it has been observed that zero-valent metals, with a higher specific surface, exert a more significant downregulation effect on carbohydrate metabolism. It has also been observed that spherical- or sheet-shaped nanoparticles as well as particles without oxygen functional groups positively affect the upregulation of amino acid metabolism. These results can enhance our understanding of the prediction of environmental risks and provide insights into the development of eco-friendly nanoparticles.

### 4.5. Additives in Consumer Products 

Several additives, most of which contain harmful substances, are present in consumer products [164]. For example, polybrominated diphenyl ethers (PBDEs) are frequently used in many consumer products because they can prevent the spread of fires [165]. These PBDEs, which have been observed in various environments (e.g., air, ocean, and soil) as well as living organisms, are stable compounds that can remain in the environment for long periods and induce toxic effects similar to endocrine-disrupting chemicals. Specifically, metabolic disorders induced by exposure to 2,2′,4,4′-tetrabromodiphenyl ether (BDE-47) in a Drosophila model have been studied [166]. In the previous study, metabolomics investigations were performed using a combination of LC–MS and GC–MS to identify a comprehensive range of metabolites. It was observed that 48 metabolites involved in tryptophan metabolism, phenylalanine metabolism, and purine metabolism, were significantly affected. Further quantification revealed that exposure to BDE-47 alters kynurenine metabolism and induces oxidative stress as well as methylation imbalance, which is a sign of the progression of Parkinson’s Disease [167].

BPA is frequently used in daily products, such as in beverage packaging, paper coatings, and flame retardants [168,169]. Given that BPA has been used for a long time, it has been discharged into the environment in large quantities, leading to human exposure. Therefore, interest in its toxic effects has increased over the years, and several studies have been conducted on this topic. In a recent study, the toxic effect of BPA on the aquatic midge, *Chironomus riparius*, was evaluated [170]. Through reproduction assays, DNA damage assays, and integrated epigenetic and metabolomics approaches, the stress response mechanisms that occur in this aquatic organism following exposure to BPA were clarified with a result showing that BPA induces reproductive failure and increase DNA damage. Additionally, NMR-based metabolomics confirmed perturbations in energy and methionine metabolism. A combination of metabolomics and epigenetic studies revealed that BPA could induce global DNA methylation and perturbation of key metabolic pathways. Therefore, to reduce environmental risks induced by environmental pollutants, it is necessary to carefully control the levels of consumer product additives.

**Table 3:** Summary of perturbation of metabolic pathways in model organisms in response to environmental toxicants.

	Organisms	Environmental Toxicants	Experimental Conditions	Toxic Mechanisms	Biochemical Assays	References
Pharmaceuticals and personal care products	*Danio rerio*	Clarithromycin Florfenicol Sulfamethazine	Adult fish (n = 30) 0.1 mg/L for 72 h Extraction with Bligh-Dyer LC-QToF-MS	Dysregulation of choline, guanosine, and ADP	Impaired swimming behavior	De Sotto et al. [146]
		Triclsoan (1, 30 and 300 μg/L) Methyl triclosan (MTCS) (0.5, 10 and 400 μg/L)	50 embryos (n = 6) for 96 h Extraction with acetonitrile: isopropanol: water (3:3:2) GC-MS	Dysregulation of energy metabolism, nitrogen metabolism, and fatty acid synthesis	Dysregulation of eight genes related to energy metabolism, nitrogen metabolism, and fatty acid synthesis	Fu et al. [147]
	*Gammarus pulex*	Propranolol (100, 153 mg/L) Triclosan (0.1, 0.3 mg/L) Nimesulide (0.5, 1.4 mg/L)	Adult G. pulex (2, 6, 24 h) (approx. 100 specimens, n = 4) Extraction with 90% MeOH LC-Orbitrap-MS	Possible alterations of protein syntehsis and oxidative stress	Three pharmaceuticals affected 23 functional pathways	Sheikholeslami et al. [171]
	*Mytilus galloprovincialis*	Diclofenac (100 μg/L)	3 mussels (n = 6) for 7 days Extraction with water: methanol: dichlromethane LC-Orbitrap-MS	Dysregulation of tyrosine and tryptophan metabolism	Potential risk of osmoregulation and reprodution	Bonnefille et al. [172]
		Sulfamethoxazole	10 mussels exposed 4 days Extraction with metanol/water (1:2), clean up with SPE LC-QTrap-MS	Significant change in four amino acids, Benzoic acid, and Inosine	Perturbation of osmoregulation, energy metabolism, and organoleptic properties	Serra-Compte et al. [173]
	*Caenorhabditis elegans*	Triclosan (0.1 and 1 mg/L)	Adult worms exposed 24 h Extraction with 80% MeOH GC-MS after silylation	Significantly affected amino aicds, tricyclic acid intermediates, carbohydrates and poly amines.	Decreased lifespan, reproduction, and locomotion. Increased oxidative stress	Kim et al. [150]
Pesticides	*Danio rerio*	Dieldrin (16 or 163.5 ng/g)	Adult fish Extraction with acetone: hexane (5:2), reconstitution with ACN, clean up with SPE, GC-MS/MS	Dieldrin altered composition and function of intestinal microbiome	No change in body mass, growth rate or histopathology.	Hua et al. [174]
		Isocarbophos (50 and 200 μg/L)	Adult fish exposed 4 days Extraction with 20% MeOH ^1^H-NMR analyasis	Significant alteration with energy related metabolism (lactate, alanine, and creatin)	Significant down-regulation of antioxidant enzyme activity. Accumulation of Isocarbophos in zebrafish	Jia et al. [175]
	*Oryza sativa*	Butachlor (3.148 kg/a.i.ha) Chlorpyrifos (1.440 kg/a.i.ha) Tricyclazole (0.607 kg/a.i.ha)	10 mg of dried leaves Extraction with methanol: chloroform: water (5:2:2) GC-MS after silylation	Signifcantly affected TCA cyle, amino acid, and fatty acid metabolism.	Fifferentially expressed genes starch-sucrose distribution, protein contents and photosynthesis	Liu et al. [156]
	*Eisenia fetida*	Sulfoxaflor (0/2 mg/kg)	Earthworms exposed 14 days Extractions with methanol: acetonitrile: water (2:2:1) LC-Orbitrap-MS	Sulfoxaflor altered carbohydrates, TCA cycle, pyrimidine purine, and some amino acids	Oxidative damage by sulfoxaflor was confirmed by SOD, CAT, GST, and MDA assay	Fang et al. [153]
		Imidacloprid Dinotefuran	Earthworms exposed 7, 14, 21, 28 days Extration with acetontirile: methanol:water (1:2:1) LC-QToF-MS	Disturbance of TCA/Urea cycle, energy production and oxidative stress.	Alteration of activity acetylcholineesterase, superoixde dismutase, and catalase	Zhang et al. [176]
	*Caenorhabditis elegans*	Atrazine (4 mg/L)	10,000 worms (n = 5) for 48 h Extractions with methanol: acetonitrile: water (2:2:1) LC-Orbitrap-MS	Perturbation of glycolysis, gluconeogenesis, and phosphatidylcholine metabolism	Increased oxidative stress and disrput ATP synthesis. Reduction of reproduction, locomotion and brood size	Yin et al. [177]
Nanoparticles	*Oreochromis mossambicus*	100 nm polystyrene (20 mg/L)	20 fish for 7 days Extractions with methanol: acetonitrile: water (2:2:1) LC-QToF-MS	Disorder of energy, amino acid, and lipid metabolism.	Damage of feeding and sensing behavior and signaling disorder	Pang et al. [178]
	*Danio rerio*	polypropylene fibers (10 and 100 μg/L)	Adult fish for 21 days Intestines were extracted with methanol: water (4:1) UPLC-MS	Up-regulation of glycerophospholipids metabolism and down-regulation of fatty acyls metabolism.	Intestinal damage, nutritional deficiency, and oxidative stress were induced by microplastic fibers	Zhao et al. [179]
	*Cyprinus carpio*	Silver nanoparticle (0.1, 0.5, 1 and 2 mg/L)	10 adult fish for 24~96 h Fish gills were extracted with methanol: water (4:1) UPLC-QToF-MS	Inhibition of TCA cycle. Perturbation of lipid metabolism.	Induced epithelial hyperplasis of gill. Perturbation of genes in asparatate metabolism pathways	Xiang et al. [180]
	*Poterioochromonas malhamensis*	Silver nanoparticle (1 mg/L)	Algae for 2 and 24 h Extractions with methanol: water (4:1) LC-QqQ-MS	Perturbation of amino acids, nucleobases, sugars, and fatty acids metabolism.	Increased level of ROS and decrease of the photosynthetic efficiency	Liu et al. [181]
	*Eisenia fetida*	TiO_2_ nanoparticles (5, 50, 500 mg/kg)	Earthworms for 120 days Extractions with chloroform: water: methanol (2:2:5) GC-MS after silylation	Alteration of glutathione and starch, sucrose metabolism.	Decreased GSH/GSSG ratio. Slight increase in ROS level. Alteration of genes in TGF-beta singaling pathway	Zhu et al. [182]
	*Enchytraeus crypticus*	Silver nanoparticles (60–102 mg/kg) Silver ions (45–60 mg/kg)	Worms (n = 5) for 7 and 14 days Extraction with methanol LC-Orbitrap-MS	Alteration of phenylalanine, histidine, lipid, and energy metabolism.	Activation of cellular iron ion homeostasis, tyrosine catabolism, glycosylation, and stress response	Maria et al. [183]
Consumer Products Additives	*Drosophila melanogaster*	BDE-47 (2, 10 or 50 μM)	5 flies for 30 days Extraction with methanol LC-Orbitrap-MS Extraction with methanol: water (4:1) GC-MS after silylation	Perturbation of metabolites involved in tryptophan, phenlyalanine, and purine metabolism	Decreased ratio of SAM/SAH and GSH/GSSG. Imbalance of kynurenine metabolism and oxidative stress	Ji et al. [166]
	*Eisenia fetida*	BDE-47 BDE-209 (10, 50, 100 and 200 mg/kg)	3 earthworms (n = 6) for 14 days Extraction with water ^1^H-NMR analysis	Increase of lactate, glutamate, betaine, leucine and lysine. Decrease of fumarate and glycine.	Toxic effects by disturbing osmo regulation, energy metabolism, nerve activities, and TCA cycle	Liang et al. [184]
	*Danio rerio*	Bisphenol A (4.4, 8.8, 17.5 μM)	20 zebrafish embryos (n = 6) Extraction with methanol: water: chloroform LC-QToF-MS	Perturbation of amino acids, prostaglandin, folate, ascorbate and nucleotide metabolic pathways.	Altered gene expression with estrogenic, CYP450 enzyme, tissue development and cell proliferation	Ortiz-Villanueva et al. [185]
	*Rattus norvegicus*	Bisphenol A Bisphenol S (50 μg/kg)	Rat plasma (n = 14) Extraction with methanol: water (4:1) LC-Orbitrap-MS	BPA exposure decreased citric acid, oxoglutaric acid, and malic acid. While BPS decreased poly unsaturated fatty acid.	Toxic effects of endocrine disruption, cytotoxicity, and genotoxicity	Mao et al. [186]
	*Mytilus coruscus*	Phthalates (0.04, 0.40, 1.00 mg/L)	4 mussels for 7 days. Extraction with methanol LC-QToF-MS	Significant changes in amino acids, lipids, energy storage compounds, osmolytes and neurotransmittes.	Activation of antioxidant defense system	Gu et al. [187]

## 5. Future Perspective

Metabolomics plays an important role in ecotoxicology and can be used to evaluate the effects of environmental pollutants, such as xenobiotics, in living organisms. This approach offers the possibility of identifying perturbations in metabolite levels and clarifying associated toxic mechanisms based on the results obtained. In recent years, there has been an increase in the number of environmental metabolomic studies on the toxic effects of pharmaceuticals, pesticides, and nanoparticles in model organisms. These studies have led to significant progress in environmental science. The risk assessment of environmental pollutants has also raised awareness in this regard. However, owing to differences in their chemical properties, profiling all the metabolites present in a sample using a single method is challenging. Specifically, the identification of metabolites is not straightforward when an untargeted approach is used. Nonetheless, it is now possible to detect a wide range of metabolites owing to advancements in technology; for examples, the high sensitivity of MS enables the identification of trace amounts of metabolites. In particular, HRMS allows the accurate measurement of *m*/*z* values even to four decimal points, and advanced peak detection processes have rendered the deconvolution of the mass spectrum easy. Given that well-established libraries have been established by a number of research groups, several methods for the identification of metabolites are now available. Notwithstanding, standardization of the metabolomics workflow is essential to take full advantage of these techniques. In addition, several factors that are determined during pre-analytical, analytical, and post-analytical steps can affect the results of metabolite detection, identification, and quantitation. Moreover, major groups in this field have reported standard requirements when reporting environmental metabolomics work [188]. This report provides information guidelines, such as the characteristics of the model organism, a description of environmental conditions, and detailed explanation of the experimental procedures. Therefore, the need for standardization, which has led to active discussions on the standardization of the overall metabolomics workflow, has been highlighted. This is an important factor in ensuring high-quality environmental metabolomic results. Given that using a multi-omics approach, altered levels of metabolites and information on their related genes, proteins, and transcription factors can be acquired, functional interpretation can be improved via the integration of multi-omics approaches, such as transcriptomics and proteomics, combined with metabolomics results. These approaches can enhance the understanding of the comprehensive toxic mechanisms of environmental pollutants and can be combined with phenotypic results to achieve a comprehensive understanding of the underlying toxic mechanisms. Finally, the relationships between environmental pollutants and their synergistic effects can also be studied by analyzing the behavior and toxic effects of pollutants when model organisms are simultaneously exposed to several pollutants. As various types of contaminants exist in the environment, they possibly interact with each other, adopting forms that are different from those they originally had. Therefore, it is important to study the effects of coexistence. With advances in metabolomics-related technologies, studies on various environmental pollutants can be conducted. However, there are challenges in the comprehensive understanding of interactions in a mixture of pollutants. Researchers are actively developing approaches that can help to understand the joint effects of chemical mixtures [189]. Applying these tools with advanced metabolomics studies will help to elucidate changes in pollutant absorption rates. Nonetheless, the effects of toxicity due to interactions between environmental pollutants require further study. Given that several environmental pollutants flow into the ocean as well as the soil, their coexistence is highly probable. Thus, it is important to perform in-depth studies to monitor the interaction effects of these pollutants.

## Figures and Tables

**Table 2:** Instrument for MS-based metabolomics approach.

Platform	Separation	Mobile Phase	Metabolic Scope	Limitation
RPLC-MS	C18 column	Water → ACN or MeOH	Polar and medium polar	- Not for the samples, including non-polar metabolites such as triacylglycerol since ACN or MeOH is not strong enough to elute non-polar compounds - Very polar metabolites could be eluted simultaneously in early retention time
HILIC-MS	Amide, Silica	ACN → Water	Very polar metabolite	- Much longer equilibration time needed compared to RPLC - Detectable metabolites greatly depend on the pH of mobile phase
IP-RPLC-MS	C18	Water → ACN or MeOH with ion paring agent	Very polar metabolite	- Permanent contamination of LC system and ion source with ion paring reagents.
GC-MS	Polysiloxane	Helium or Nitrogen	Volatile metabolites	- Derivatization is needed in the sample preparation step
CE-MS	Fused-silica Capillary with polymer	Water, ACN, MeOH	Neutral, anionic, cationic metabolite	- Low migration time repeatablity - Poor detection sensitivity

## Data Availability

Not applicable.

## References

[1] Liang Y., Tan Q., Song Q., Li J. (2021). An analysis of the plastic waste trade and management in Asia. Waste Manag..

[2] Blettler M.C., Wantzen K.M. (2019). Threats underestimated in freshwater plastic pollution: Mini-review. Water Air Soil Pollut..

[3] Mezzelani M., Nardi A., Bernardini I., Milan M., Peruzza L., d’Errico G., Fattorini D., Gorbi S., Patarnello T., Regoli F. (2021). Environmental pharmaceuticals and climate change: The case study of carbamazepine in M. galloprovincialis under ocean acidification scenario. Environ. Int..

[4] Balmer J.E., Morris A.D., Hung H., Jantunen L., Vorkamp K., Rigét F., Evans M., Houde M., Muir D.C. (2019). Levels and trends of current-use pesticides (CUPs) in the arctic: An updated review, 2010–2018. Emerg. Contam..

[5] Yang L., Zhou Y., Shi B., Meng J., He B., Yang H., Yoon S.J., Kim T., Kwon B.-O., Khim J.S. (2020). Anthropogenic impacts on the contamination of pharmaceuticals and personal care products (PPCPs) in the coastal environments of the Yellow and Bohai seas. Environ. Int..

[6] Jones O.A.H., Green P.G., Voulvoulis N., Lester J.N. (2007). Questioning the Excessive Use of Advanced Treatment to Remove Organic Micropollutants from Wastewater. Environ. Sci. Technol..

[7] Huang Q., Liu Y., Chen Y., Fang C., Chi Y., Zhu H., Lin Y., Ye G., Dong S. (2018). New insights into the metabolism and toxicity of bisphenol A on marine fish under long-term exposure. Environ. Pollut..

[8] Zhang X., Wen K., Ding D., Liu J., Lei Z., Chen X., Ye G., Zhang J., Shen H., Yan C. (2021). Size-dependent adverse effects of microplastics on intestinal microbiota and metabolic homeostasis in the marine medaka (Oryzias melastigma). Environ. Int..

[9] Cong Y., Jin F., Wang J., Mu J. (2017). The embryotoxicity of ZnO nanoparticles to marine medaka, Oryzias melastigma. Aquat. Toxicol..

[10] Sun D., Chen Q., Zhu B., Lan Y., Duan S. (2020). Long-term exposure to benzo [a] pyrene affects sexual differentiation and embryos toxicity in three generations of marine Medaka (Oryzias melastigma). Int. J. Environ. Res. Public Health.

[11] Hansen B.H., Sørensen L., Størseth T.R., Altin D., Gonzalez S.V., Skancke J., Rønsberg M.U., Nordtug T. (2020). The use of PAH, metabolite and lipid profiling to assess exposure and effects of produced water discharges on pelagic copepods. Sci. Total Environ..

[12] Aru V., Balling Engelsen S., Savorani F., Culurgioni J., Sarais G., Atzori G., Cabiddu S., Marincola F.C. (2017). The effect of season on the metabolic profile of the European clam Ruditapes decussatus as studied by 1H-NMR spectroscopy. Metabolites.

[13] Wang L., Huang X., Sun W., Too H.Z., Laserna A.K.C., Li S.F.Y. (2020). A global metabolomic insight into the oxidative stress and membrane damage of copper oxide nanoparticles and microparticles on microalga Chlorella vulgaris. Environ. Pollut..

[14] Hu B., Shao S., Ni H., Fu Z., Hu L., Zhou Y., Min X., She S., Chen S., Huang M. (2020). Current status, spatial features, health risks, and potential driving factors of soil heavy metal pollution in China at province level. Environ. Pollut..

[15] Manisalidis I., Stavropoulou E., Stavropoulos A., Bezirtzoglou E. (2020). Environmental and health impacts of air pollution: A review. Front. Public Health.

[16] Yao X., Zhang F., Qiao Z., Yu H., Sun S., Li X., Zhang J., Jiang X. (2020). Toxicity of thifluzamide in earthworm (Eisenia fetida). Ecotoxicol. Environ. Saf..

[17] Zhu L., Li B., Wu R., Li W., Wang J., Wang J., Du Z., Juhasz A., Zhu L. (2020). Acute toxicity, oxidative stress and DNA damage of chlorpyrifos to earthworms (Eisenia fetida): The difference between artificial and natural soils. Chemosphere.

[18] Kim H.M., Lee D.-K., Long N.P., Kwon S.W., Park J.H. (2019). Uptake of nanopolystyrene particles induces distinct metabolic profiles and toxic effects in Caenorhabditis elegans. Environ. Pollut..

[19] Kim H.M., Long N.P., Min J.E., Anh N.H., Kim S.J., Yoon S.J., Kwon S.W. (2020). Comprehensive phenotyping and multi-omic profiling in the toxicity assessment of nanopolystyrene with different surface properties. J. Hazard. Mater..

[20] Geng N., Song X., Cao R., Luo Y., Mila A., Cai Z., Yu K., Gao Y., Ni Y., Zhang H. (2021). The effect of toxic components on metabolomic response of male SD rats exposed to fine particulate matter. Environ. Pollut..

[21] Du X., Zeng X., Pan K., Zhang J., Song L., Zhou J., Chen R., Xie Y., Sun Q., Zhao J. (2020). Metabolomics analysis of urine from healthy wild type mice exposed to ambient PM2.5. Sci. Total Environ..

[22] Blaženović I., Kind T., Sa M.R., Ji J., Vaniya A., Wancewicz B., Roberts B.S., Torbašinović H., Lee T., Mehta S.S. (2019). Structure annotation of all mass spectra in untargeted metabolomics. Anal. Chem..

[23] Long N.P., Nghi T.D., Kang Y.P., Anh N.H., Kim H.M., Park S.K., Kwon S.W. (2020). Toward a standardized strategy of clinical metabolomics for the advancement of precision medicine. Metabolites.

[24] Labine L.M., Simpson M.J. (2020). The use of nuclear magnetic resonance (NMR) and mass spectrometry (MS)–based metabolomics in environmental exposure assessment. Curr. Opin. Environ. Sci. Health.

[25] Gil-Solsona R., Álvarez-Muñoz D., Serra-Compte A., Rodríguez-Mozaz S. (2021). (Xeno) Metabolomics for the evaluation of aquatic organism’s exposure to field contaminated water. Trends Environ. Anal. Chem..

[26] Kovacevic V., Simpson M.J. (2020). Fundamentals of environmental metabolomics. Environmental Metabolomics.

[27] Liang D., Moutinho J.L., Golan R., Yu T., Ladva C.N., Niedzwiecki M., Walker D.I., Sarnat S.E., Chang H.H., Greenwald R. (2018). Use of high-resolution metabolomics for the identification of metabolic signals associated with traffic-related air pollution. Environ. Int..

[28] Matich E.K., Soria N.G.C., Aga D.S., Atilla-Gokcumen G.E. (2019). Applications of metabolomics in assessing ecological effects of emerging contaminants and pollutants on plants. J. Hazard. Mater..

[29] Xu T., Zhang M., Hu J., Li Z., Wu T., Bao J., Wu S., Lei L., He D. (2017). Behavioral deficits and neural damage of Caenorhabditis elegans induced by three rare earth elements. Chemosphere.

[30] Pan H., Zhang X., Ren B., Yang H., Ren Z., Wang W. (2017). Toxic assessment of cadmium based on online swimming behavior and the continuous AChE activity in the gill of zebrafish (Danio rerio). Water Air Soil Pollut..

[31] Duan J., Hu H., Zhang Y., Feng L., Shi Y., Miller M.R., Sun Z. (2017). Multi-organ toxicity induced by fine particulate matter PM2. 5 in zebrafish (Danio rerio) model. Chemosphere.

[32] Li S.-W., How C.M., Liao V.H.-C. (2018). Prolonged exposure of di (2-ethylhexyl) phthalate induces multigenerational toxic effects in Caenorhabditis elegans. Sci. Total Environ..

[33] Ge J., Xiao Y., Chai Y., Yan H., Wu R., Xin X., Wang D., Yu X. (2018). Sub-lethal effects of six neonicotinoids on avoidance behavior and reproduction of earthworms (Eisenia fetida). Ecotoxicol. Environ. Saf..

[34] Iummato M.M., Sabatini S.E., Cacciatore L.C., Cochón A.C., Cataldo D., de Molina M.d.C.R., Juárez Á.B. (2018). Biochemical responses of the golden mussel Limnoperna fortunei under dietary glyphosate exposure. Ecotoxicol. Environ. Saf..

[35] Nogueira A.F., Pinto G., Correia B., Nunes B. (2019). Embryonic development, locomotor behavior, biochemical, and epigenetic effects of the pharmaceutical drugs paracetamol and ciprofloxacin in larvae and embryos of Danio rerio when exposed to environmental realistic levels of both drugs. Environ. Toxicol..

[36] Qu M., Wang D. (2020). Toxicity comparison between pristine and sulfonate modified nanopolystyrene particles in affecting locomotion behavior, sensory perception, and neuronal development in Caenorhabditis elegans. Sci. Total Environ..

[37] Hemalatha D., Rangasamy B., Nataraj B., Maharajan K., Narayanasamy A., Ramesh M. (2020). Transcriptional, biochemical and histological alterations in adult zebrafish (Danio rerio) exposed to benzotriazole ultraviolet stabilizer-328. Sci. Total Environ..

[38] Sousa A.P., Nunes B. (2020). Standard and biochemical toxicological effects of zinc pyrithione in Daphnia magna and Daphnia longispina. Environ. Toxicol. Pharmacol..

[39] Yuan N., Pei Y., Bao A., Wang C. (2020). The Physiological and Biochemical Responses of Daphnia magna to Dewatered Drinking Water Treatment Residue. Int. J. Environ. Res. Public Health.

[40] Gowri S., Thangaraj R. (2020). Studies on the toxic effects of agrochemical pesticide (Monocrotophos) on physiological and reproductive behavior of indigenous and exotic earthworm species. Int. J. Environ. Health Res..

[41] Cong Y., Wang Y., Zhang M., Jin F., Mu J., Li Z., Wang J. (2021). Lethal, behavioral, growth and developmental toxicities of alkyl-PAHs and non-alkyl PAHs to early-life stage of brine shrimp, Artemia parthenogenetica. Ecotoxicol. Environ. Saf..

[42] Yang W., Gao P., Ma G., Huang J., Wu Y., Wan L., Ding H., Zhang W. (2021). Transcriptome analysis of the toxic mechanism of nanoplastics on growth, photosynthesis and oxidative stress of microalga Chlorella pyrenoidosa during chronic exposure. Environ. Pollut..

[43] Fraga-Corral M., Carpena M., Garcia-Oliveira P., Pereira A., Prieto M., Simal-Gandara J. (2020). Analytical metabolomics and applications in health, environmental and food science. Crit. Rev. Anal. Chem..

[44] Percival B.C., Grootveld M., Gibson M., Osman Y., Molinari M., Jafari F., Sahota T., Martin M., Casanova F., Mather M.L. (2019). Low-field, benchtop NMR spectroscopy as a potential tool for point-of-care diagnostics of metabolic conditions: Validation, protocols and computational models. High-Throughput.

[45] Locci E., Bazzano G., Chighine A., Locco F., Ferraro E., Demontis R., d’Aloja E. (2020). Forensic NMR metabolomics: One more arrow in the quiver. Metabolomics.

[46] Grootveld M., Percival B., Gibson M., Osman Y., Edgar M., Molinari M., Mather M.L., Casanova F., Wilson P.B. (2019). Progress in low-field benchtop NMR spectroscopy in chemical and biochemical analysis. Anal. Chim. Acta.

[47] Crook A.A., Powers R. (2020). Quantitative NMR-Based Biomedical Metabolomics: Current Status and Applications. Molecules.

[48] Simpson A.J., Simpson M.J., Soong R. (2018). Environmental nuclear magnetic resonance spectroscopy: An overview and a primer. Anal. Chem..

[49] Augustijn D., de Groot H.J., Alia A. (2021). HR-MAS NMR applications in plant metabolomics. Molecules.

[50] de Souza L.P., Alseekh S., Naake T., Fernie A. (2019). Mass Spectrometry-Based Untargeted Plant Metabolomics. Curr. Protoc. Plant Biol..

[51] Cajka T., Fiehn O. (2016). Toward merging untargeted and targeted methods in mass spectrometry-based metabolomics and lipidomics. Anal. Chem..

[52] Longnecker K., Futrelle J., Coburn E., Soule M.C.K., Kujawinski E.B. (2015). Environmental metabolomics: Databases and tools for data analysis. Mar. Chem..

[53] Kusano M., Yang Z., Okazaki Y., Nakabayashi R., Fukushima A., Saito K. (2015). Using metabolomic approaches to explore chemical diversity in rice. Mol. Plant.

[54] Zhang W., Ramautar R. (2021). CE-MS for metabolomics: Developments and applications in the period 2018–2020. Electrophoresis.

[55] Stettin D., Poulin R.X., Pohnert G. (2020). Metabolomics Benefits from Orbitrap GC–MS—Comparison of Low-and High-Resolution GC–MS. Metabolites.

[56] Scott E.R., Li X., Kfoury N., Morimoto J., Han W.-Y., Ahmed S., Cash S.B., Griffin T.S., Stepp J.R., Robbat A. (2019). Interactive effects of drought severity and simulated herbivory on tea (Camellia sinensis) volatile and non-volatile metabolites. Environ. Exp. Bot..

[57] Ren J.-L., Zhang A.-H., Kong L., Wang X.-J. (2018). Advances in mass spectrometry-based metabolomics for investigation of metabolites. RSC Adv..

[58] Beale D.J., Pinu F.R., Kouremenos K.A., Poojary M.M., Narayana V.K., Boughton B.A., Kanojia K., Dayalan S., Jones O.A., Dias D.A. (2018). Review of recent developments in GC–MS approaches to metabolomics-based research. Metabolomics.

[59] Liebeke M., Puskás E. (2019). Drying enhances signal intensities for global GC–MS metabolomics. Metabolites.

[60] Hassan H.A., Ammar N.M., Serag A., Shaker O.G., El Gendy A.N., Abdel-Hamid A.-H.Z. (2020). Metabolomics driven analysis of obesity-linked colorectal cancer patients via GC-MS and chemometrics: A pilot study. Microchem. J..

[61] Cui L., Lu H., Lee Y.H. (2018). Challenges and emergent solutions for LC-MS/MS based untargeted metabolomics in diseases. Mass Spectrom. Rev..

[62] Blaženović I., Kind T., Ji J., Fiehn O. (2018). Software tools and approaches for compound identification of LC-MS/MS data in metabolomics. Metabolites.

[63] Chetwynd A.J., David A. (2018). A review of nanoscale LC-ESI for metabolomics and its potential to enhance the metabolome coverage. Talanta.

[64] Xu Y.-F., Lu W., Rabinowitz J.D. (2015). Avoiding misannotation of in-source fragmentation products as cellular metabolites in liquid chromatography–mass spectrometry-based metabolomics. Anal. Chem..

[65] Xian F., Hendrickson C.L., Marshall A.G. (2012). High resolution mass spectrometry. Anal. Chem..

[66] Kind T., Tsugawa H., Cajka T., Ma Y., Lai Z., Mehta S.S., Wohlgemuth G., Barupal D.K., Showalter M.R., Arita M. (2018). Identification of small molecules using accurate mass MS/MS search. Mass Spectrom. Rev..

[67] Yuan M., Breitkopf S.B., Yang X., Asara J.M. (2012). A positive/negative ion–switching, targeted mass spectrometry–based metabolomics platform for bodily fluids, cells, and fresh and fixed tissue. Nat. Protoc..

[68] Ye Y., Zhan H., Yu X., Li J., Wang X., Xie Z. (2021). Detection of organosulfates and nitrooxy-organosulfates in Arctic and Antarctic atmospheric aerosols, using ultra-high resolution FT-ICR mass spectrometry. Sci. Total Environ..

[69] Naz S., Gallart-Ayala H., Reinke S.N., Mathon C., Blankley R., Chaleckis R., Wheelock C.E. (2017). Development of a liquid chromatography–high resolution mass spectrometry metabolomics method with high specificity for metabolite identification using all ion fragmentation acquisition. Anal. Chem..

[70] Cajka T., Smilowitz J.T., Fiehn O. (2017). Validating quantitative untargeted lipidomics across nine liquid chromatography–high-resolution mass spectrometry platforms. Anal. Chem..

[71] Dudzik D., Barbas-Bernardos C., García A., Barbas C. (2018). Quality assurance procedures for mass spectrometry untargeted metabolomics. a review. J. Pharm. Biomed. Anal..

[72] Bonvallot N., David A., Chalmel F., Chevrier C., Cordier S., Cravedi J.-P., Zalko D. (2018). Metabolomics as a powerful tool to decipher the biological effects of environmental contaminants in humans. Curr. Opin. Toxicol..

[73] Gehrke S., Reisz J.A., Nemkov T., Hansen K.C., D’Alessandro A. (2017). Characterization of rapid extraction protocols for high-throughput metabolomics. Rapid Commun. Mass Spectrom..

[74] Mastroianni G., Scognamiglio M., Russo C., Fiorentino A., Lavorgna M. (2020). Environmental Metabolomics: A Powerful Tool to Investigate Biochemical Responses to Drugs in Nontarget Organisms. Fate and Effects of Anticancer Drugs in the Environment.

[75] Lin C.Y., Wu H., Tjeerdema R.S., Viant M.R. (2007). Evaluation of metabolite extraction strategies from tissue samples using NMR metabolomics. Metabolomics.

[76] Yan S.-C., Chen Z.-F., Zhang H., Chen Y., Qi Z., Liu G., Cai Z. (2020). Evaluation and optimization of sample pretreatment for GC/MS-based metabolomics in embryonic zebrafish. Talanta.

[77] Geier F.M., Want E.J., Leroi A.M., Bundy J.G. (2011). Cross-platform comparison of Caenorhabditis elegans tissue extraction strategies for comprehensive metabolome coverage. Anal. Chem..

[78] Chen S., Hoene M., Li J., Li Y., Zhao X., Häring H.-U., Schleicher E.D., Weigert C., Xu G., Lehmann R. (2013). Simultaneous extraction of metabolome and lipidome with methyl tert-butyl ether from a single small tissue sample for ultra-high performance liquid chromatography/mass spectrometry. J. Chromatogr. A.

[79] Medina J., van der Velpen V., Teav T., Guitton Y., Gallart-Ayala H., Ivanisevic J. (2020). Single-Step Extraction Coupled with Targeted HILIC-MS/MS Approach for Comprehensive Analysis of Human Plasma Lipidome and Polar Metabolome. Metabolites.

[80] Ibáñez C., Simó C., Palazoglu M., Cifuentes A. (2017). GC-MS based metabolomics of colon cancer cells using different extraction solvents. Anal. Chim. Acta.

[81] Moros G., Chatziioannou A.C., Gika H.G., Raikos N., Theodoridis G. (2017). Investigation of the derivatization conditions for GC–MS metabolomics of biological samples. Bioanalysis.

[82] He Z., Luo Q., Liu Z., Gong L. (2020). Extensive evaluation of sample preparation workflow for gas chromatography-mass spectrometry-based plasma metabolomics and its application in rheumatoid arthritis. Anal. Chim. Acta.

[83] Poole C.F. (2003). New trends in solid-phase extraction. TrAC Trends Anal. Chem..

[84] Tang D.Q., Zou L., Yin X.X., Ong C.N. (2016). HILIC-MS for metabolomics: An attractive and complementary approach to RPLC-MS. Mass Spectrom. Rev..

[85] Spagou K., Tsoukali H., Raikos N., Gika H., Wilson I.D., Theodoridis G. (2010). Hydrophilic interaction chromatography coupled to MS for metabonomic/metabolomic studies. J. Sep. Sci..

[86] Zhou B., Xiao J.F., Tuli L., Ressom H.W. (2012). LC-MS-based metabolomics. Mol. BioSyst..

[87] Contrepois K., Jiang L., Snyder M. (2015). Optimized analytical procedures for the untargeted metabolomic profiling of human urine and plasma by combining hydrophilic interaction (HILIC) and reverse-phase liquid chromatography (RPLC)–mass spectrometry. Mol. Cell. Proteom..

[88] Schwaiger M., Schoeny H., El Abiead Y., Hermann G., Rampler E., Koellensperger G. (2019). Merging metabolomics and lipidomics into one analytical run. Analyst.

[89] Liu Z., Ezernieks V., Rochfort S., Cocks B. (2018). Comparison of methylation methods for fatty acid analysis of milk fat. Food Chem..

[90] Ooi M., Nishiumi S., Yoshie T., Shiomi Y., Kohashi M., Fukunaga K., Nakamura S., Matsumoto T., Hatano N., Shinohara M. (2011). GC/MS-based profiling of amino acids and TCA cycle-related molecules in ulcerative colitis. Inflamm. Res..

[91] Lu Y., Gao K., Li X., Tang Z., Xiang L., Zhao H., Fu J., Wang L., Zhu N., Cai Z. (2019). Mass spectrometry-based metabolomics reveals occupational exposure to per-and polyfluoroalkyl substances relates to oxidative stress, fatty acid β-oxidation disorder, and kidney injury in a manufactory in China. Environ. Sci. Technol..

[92] Lebedev A.T., Polyakova O.V., Mazur D.M., Artaev V.B. (2013). The benefits of high resolution mass spectrometry in environmental analysis. Analyst.

[93] Feith A., Teleki A., Graf M., Favilli L., Takors R. (2019). HILIC-enabled 13C metabolomics strategies: Comparing quantitative precision and spectral accuracy of QTOF high-and QQQ low-resolution mass spectrometry. Metabolites.

[94] Cao G., Song Z., Hong Y., Yang Z., Song Y., Chen Z., Chen Z., Cai Z. (2020). Large-scale targeted metabolomics method for metabolite profiling of human samples. Anal. Chim. Acta.

[95] Reisz J.A., Zheng C., D’Alessandro A., Nemkov T. (2019). Untargeted and semi-targeted lipid analysis of biological samples using mass spectrometry-based metabolomics. High-Throughput Metabolomics.

[96] Yuan P., Dong M., Lei H., Xu G., Chen G., Song Y., Ma J., Cheng L., Zhang L. (2020). Targeted metabolomics reveals that 2, 3, 7, 8-tetrachlorodibenzofuran exposure induces hepatic steatosis in male mice. Environ. Pollut..

[97] Domingo-Almenara X., Montenegro-Burke J.R., Ivanisevic J., Thomas A., Sidibé J., Teav T., Guijas C., Aisporna A.E., Rinehart D., Hoang L. (2018). XCMS-MRM and METLIN-MRM: A cloud library and public resource for targeted analysis of small molecules. Nat. Methods.

[98] Guo J., Huan T. (2020). Comparison of Full-Scan, Data-Dependent, and Data-Independent Acquisition Modes in Liquid Chromatography–Mass Spectrometry Based Untargeted Metabolomics. Anal. Chem..

[99] Barbier Saint Hilaire P., Rousseau K., Seyer A., Dechaumet S., Damont A., Junot C., Fenaille F. (2020). Comparative Evaluation of Data Dependent and Data Independent Acquisition Workflows Implemented on an Orbitrap Fusion for Untargeted Metabolomics. Metabolites.

[100] Hemmer S., Manier S.K., Fischmann S., Westphal F., Wagmann L., Meyer M.R. (2020). Comparison of three untargeted data processing workflows for evaluating LC-HRMS metabolomics data. Metabolites.

[101] Li Z., Lu Y., Guo Y., Cao H., Wang Q., Shui W. (2018). Comprehensive evaluation of untargeted metabolomics data processing software in feature detection, quantification and discriminating marker selection. Anal. Chim. Acta.

[102] Beger R.D., Dunn W.B., Bandukwala A., Bethan B., Broadhurst D., Clish C.B., Dasari S., Derr L., Evans A., Fischer S. (2019). Towards quality assurance and quality control in untargeted metabolomics studies. Metabolomics.

[103] Dunn W.B., Broadhurst D.I., Edison A., Guillou C., Viant M.R., Bearden D.W., Beger R.D. (2017). Quality assurance and quality control processes: Summary of a metabolomics community questionnaire. Metabolomics.

[104] Broadhurst D., Goodacre R., Reinke S.N., Kuligowski J., Wilson I.D., Lewis M.R., Dunn W.B. (2018). Guidelines and considerations for the use of system suitability and quality control samples in mass spectrometry assays applied in untargeted clinical metabolomic studies. Metabolomics.

[105] Katajamaa M., Orešič M. (2007). Data processing for mass spectrometry-based metabolomics. J. Chromatogr. A.

[106] Karaman I. (2017). Preprocessing and Pretreatment of Metabolomics Data for Statistical Analysis. Metabolomics: From Fundamentals to Clinical Applications.

[107] Nam S.L., Mata A., Dias R.P., Harynuk J.J. (2020). Towards Standardization of Data Normalization Strategies to Improve Urinary Metabolomics Studies by GC× GC-TOFMS. Metabolites.

[108] Pluskal T., Castillo S., Villar-Briones A., Orešič M. (2010). MZmine 2: Modular framework for processing, visualizing, and analyzing mass spectrometry-based molecular profile data. BMC Bioinform..

[109] Tsugawa H., Cajka T., Kind T., Ma Y., Higgins B., Ikeda K., Kanazawa M., VanderGheynst J., Fiehn O., Arita M. (2015). MS-DIAL: Data-independent MS/MS deconvolution for comprehensive metabolome analysis. Nat. Methods.

[110] Smith C.A., Want E.J., O’Maille G., Abagyan R., Siuzdak G. (2006). XCMS: Processing mass spectrometry data for metabolite profiling using nonlinear peak alignment, matching, and identification. Anal. Chem..

[111] Worley B., Powers R. (2013). Multivariate analysis in metabolomics. Curr. Metab..

[112] Ruiz-Perez D., Guan H., Madhivanan P., Mathee K., Narasimhan G. (2020). So you think you can PLS-DA?. BMC Bioinform..

[113] Vinaixa M., Samino S., Saez I., Duran J., Guinovart J.J., Yanes O. (2012). A guideline to univariate statistical analysis for LC/MS-based untargeted metabolomics-derived data. Metabolites.

[114] Smith C.A., O’Maille G., Want E.J., Qin C., Trauger S.A., Brandon T.R., Custodio D.E., Abagyan R., Siuzdak G. (2005). METLIN: A metabolite mass spectral database. Ther. Drug Monit..

[115] Fahy E., Subramaniam S., Murphy R.C., Nishijima M., Raetz C.R., Shimizu T., Spener F., van Meer G., Wakelam M.J., Dennis E.A. (2009). Update of the LIPID MAPS comprehensive classification system for lipids. J. Lipid Res..

[116] Wishart D.S., Feunang Y.D., Marcu A., Guo A.C., Liang K., Vázquez-Fresno R., Sajed T., Johnson D., Li C., Karu N. (2018). HMDB 4.0: The human metabolome database for 2018. Nucleic Acids Res..

[117] Xia J., Wishart D.S. (2010). MetPA: A web-based metabolomics tool for pathway analysis and visualization. Bioinformatics.

[118] Capela R., Garric J., Castro L.F.C., Santos M.M. (2020). Embryo bioassays with aquatic animals for toxicity testing and hazard assessment of emerging pollutants: A review. Sci. Total Environ..

[119] van de Merwe J.P., Neale P.A., Melvin S.D., Leusch F.D. (2018). In vitro bioassays reveal that additives are significant contributors to the toxicity of commercial household pesticides. Aquat. Toxicol..

[120] Heinlaan M., Kasemets K., Aruoja V., Blinova I., Bondarenko O., Lukjanova A., Khosrovyan A., Kurvet I., Pullerits M., Sihtmäe M. (2020). Hazard evaluation of polystyrene nanoplastic with nine bioassays did not show particle-specific acute toxicity. Sci. Total Environ..

[121] Hashmi M.Z., Kumar V., Varma A. (2017). Xenobiotics in the Soil Environment: Monitoring, Toxicity and Management.

[122] Abdelsalam N.A., Ramadan A.T., ElRakaiby M.T., Aziz R.K. (2020). Toxicomicrobiomics: The Human Microbiome vs. Pharmaceutical, Dietary, and Environmental Xenobiotics. Front. Pharmacol..

[123] de Oliveira M., Frihling B.E.F., Velasques J., Magalhães Filho F.J.C., Cavalheri P.S., Migliolo L. (2020). Pharmaceuticals residues and xenobiotics contaminants: Occurrence, analytical techniques and sustainable alternatives for wastewater treatment. Sci. Total Environ..

[124] Nieto-García A.J., Domínguez I., Romero-González R., Arrebola F.J., Vidal J.L.M., Frenich A.G. (2019). Automated determination of xenobiotics (pesticides, PCBs, PAHs, and PBDEs) in sediment samples applying HS-SPME-GC-HRMS. J. AOAC Int..

[125] Fatta-Kassinos D., Kalavrouziotis I.K., Koukoulakis P.H., Vasquez M. (2011). The risks associated with wastewater reuse and xenobiotics in the agroecological environment. Sci. Total Environ..

[126] Thelusmond J.-R., Strathmann T.J., Cupples A.M. (2019). Carbamazepine, triclocarban and triclosan biodegradation and the phylotypes and functional genes associated with xenobiotic degradation in four agricultural soils. Sci. Total Environ..

[127] Byrns G. (2001). The fate of xenobiotic organic compounds in wastewater treatment plants. Water Res..

[128] Bond T., Ferrandiz-Mas V., Felipe-Sotelo M., Van Sebille E. (2018). The occurrence and degradation of aquatic plastic litter based on polymer physicochemical properties: A review. Crit. Rev. Environ. Sci. Technol..

[129] Rodríguez A., Castrejón-Godínez M.L., Salazar-Bustamante E., Gama-Martínez Y., Sánchez-Salinas E., Mussali-Galante P., Tovar-Sánchez E., Ortiz-Hernández M.L. (2020). Omics approaches to pesticide biodegradation. Curr. Microbiol..

[130] Xu Y., Dai S., Meng K., Wang Y., Ren W., Zhao L., Christie P., Teng Y. (2018). Occurrence and risk assessment of potentially toxic elements and typical organic pollutants in contaminated rural soils. Sci. Total Environ..

[131] Haque E., Ward A.C. (2018). Zebrafish as a model to evaluate nanoparticle toxicity. Nanomaterials.

[132] Keller J.M., Keller E.T. (2018). The use of mature zebrafish (Danio rerio) as a model for human aging and disease. Conn’s Handbook of Models for Human Aging.

[133] Hollert H., Keiter S.H. (2015). Danio rerio as a model in aquatic toxicology and sediment research. Environ. Sci. Pollut. Res..

[134] Tkaczyk A., Bownik A., Dudka J., Kowal K., Ślaska B. (2020). Daphnia magna model in the toxicity assessment of pharmaceuticals: A review. Sci. Total Environ..

[135] Blaser R., Chadwick L., McGinnis G. (2010). Behavioral measures of anxiety in zebrafish (Danio rerio). Behav. Brain Res..

[136] Sancho E., Villarroel M., Fernández C., Andreu E., Ferrando M. (2010). Short-term exposure to sublethal tebuconazole induces physiological impairment in male zebrafish (Danio rerio). Ecotoxicol. Environ. Saf..

[137] Brenner S. (1974). The genetics of Caenorhabditis elegans. Genetics.

[138] Chen H., Wang C., Li H., Ma R., Yu Z., Li L., Xiang M., Chen X., Hua X., Yu Y. (2019). A review of toxicity induced by persistent organic pollutants (POPs) and endocrine-disrupting chemicals (EDCs) in the nematode Caenorhabditis elegans. J. Environ. Manag..

[139] Ficociello G., Inverni A., Massimi L., Buccini G., Canepari S., Uccelletti D. (2020). Assessment of the effects of atmospheric pollutants using the animal model Caenorhabditis elegans. Environ. Res..

[140] Zhang D., Gersberg R.M., Ng W.J., Tan S.K. (2014). Removal of pharmaceuticals and personal care products in aquatic plant-based systems: A review. Environ. Pollut..

[141] Wang J., Wang S. (2016). Removal of pharmaceuticals and personal care products (PPCPs) from wastewater: A review. J. Environ. Manag..

[142] Chen X., Vollertsen J., Nielsen J.L., Dall A.G., Bester K. (2015). Degradation of PPCPs in activated sludge from different WWTPs in Denmark. Ecotoxicology.

[143] Pérez-Lemus N., López-Serna R., Pérez-Elvira S.I., Barrado E. (2019). Analytical methodologies for the determination of pharmaceuticals and personal care products (PPCPs) in sewage sludge: A critical review. Anal. Chim. Acta.

[144] Dumas T., Boccard J., Gomez E., Fenet H., Courant F. (2020). Multifactorial analysis of environmental metabolomic data in ecotoxicology: Wild marine mussel exposed to wwtp effluent as a case study. Metabolites.

[145] Dumas T., Bonnefille B., Gomez E., Boccard J., Castro N.A., Fenet H., Courant F. (2020). Metabolomics approach reveals disruption of metabolic pathways in the marine bivalve Mytilus galloprovincialis exposed to a WWTP effluent extract. Sci. Total Environ..

[146] Ryan B., Medriano C.D., Cho Y., Kim H., Chung I.-Y., Seok K.-S., Song K.G., Hong S.W., Park Y., Kim S. (2017). Sub-lethal pharmaceutical hazard tracking in adult zebrafish using untargeted LC–MS environmental metabolomics. J. Hazard. Mater..

[147] Fu J., Tan Y.X.R., Gong Z., Bae S. (2020). The toxic effect of triclosan and methyl-triclosan on biological pathways revealed by metabolomics and gene expression in zebrafish embryos. Ecotoxicol. Environ. Saf..

[148] Teplova V.V., Belosludtsev K.N., Kruglov A.G. (2017). Mechanism of triclosan toxicity: Mitochondrial dysfunction including complex II inhibition, superoxide release and uncoupling of oxidative phosphorylation. Toxicol. Lett..

[149] Gillis J.D., Price G.W., Prasher S. (2017). Lethal and sub-lethal effects of triclosan toxicity to the earthworm Eisenia fetida assessed through GC–MS metabolomics. J. Hazard. Mater..

[150] Kim H.M., Long N.P., Yoon S.J., Nguyen H.T., Kwon S.W. (2019). Metabolomics and phenotype assessment reveal cellular toxicity of triclosan in Caenorhabditis elegans. Chemosphere.

[151] Yu Y., Zhu Y., Yang J., Zhu W., Zhou Z., Zhang R. (2021). Effects of Dufulin on Oxidative Stress and Metabolomic Profile of Tubifex. Metabolites.

[152] Sharma A., Kumar V., Shahzad B., Tanveer M., Sidhu G.P.S., Handa N., Kohli S.K., Yadav P., Bali A.S., Parihar R.D. (2019). Worldwide pesticide usage and its impacts on ecosystem. SN Appl. Sci..

[153] Fang S., Zhang Y., You X., Sun P., Qiu J., Kong F. (2018). Lethal toxicity and sublethal metabolic interference effects of sulfoxaflor on the earthworm (Eisenia fetida). J. Agric. Food Chem..

[154] Alengebawy A., Abdelkhalek S.T., Qureshi S.R., Wang M.-Q. (2021). Heavy metals and pesticides toxicity in agricultural soil and plants: Ecological risks and human health implications. Toxics.

[155] Elfikrie N., Ho Y.B., Zaidon S.Z., Juahir H., Tan E.S.S. (2020). Occurrence of pesticides in surface water, pesticides removal efficiency in drinking water treatment plant and potential health risk to consumers in Tengi River Basin, Malaysia. Sci. Total Environ..

[156] Liu N., Zhu L. (2020). Metabolomic and transcriptomic investigation of metabolic perturbations in *Oryza sativa* L. triggered by three pesticides. Environ. Sci. Technol..

[157] Gaylarde C.C., Neto J.A.B., da Fonseca E.M. (2020). Nanoplastics in aquatic systems-are they more hazardous than microplastics?. Environ. Pollut..

[158] Baudrimont M., Arini A., Guégan C., Venel Z., Gigault J., Pedrono B., Prunier J., Maurice L., Ter Halle A., Feurtet-Mazel A. (2020). Ecotoxicity of polyethylene nanoplastics from the North Atlantic oceanic gyre on freshwater and marine organisms (microalgae and filter-feeding bivalves). Environ. Sci. Pollut. Res..

[159] Jambeck J.R., Geyer R., Wilcox C., Siegler T.R., Perryman M., Andrady A., Narayan R., Law K.L. (2015). Plastic waste inputs from land into the ocean. Science.

[160] Mahana A., Guliy O.I., Mehta S.K. (2021). Accumulation and cellular toxicity of engineered metallic nanoparticle in freshwater microalgae: Current status and future challenges. Ecotoxicol. Environ. Saf..

[161] Chen F., Xiao Z., Yue L., Wang J., Feng Y., Zhu X., Wang Z., Xing B. (2019). Algae response to engineered nanoparticles: Current understanding, mechanisms and implications. Environ. Sci. Nano.

[162] Huang W., Wang X., Chen D., Xu E.G., Luo X., Zeng J., Huan T., Li L., Wang Y. (2021). Toxicity Mechanisms of Polystyrene Microplastics in Marine Mussels Revealed by High-Coverage Quantitative Metabolomics Using Chemical Isotope Labeling Liquid Chromatography Mass Spectrometry. J. Hazard. Mater..

[163] Li X., Ban Z., Yu F., Hao W., Hu X. (2020). Untargeted metabolic pathway analysis as an effective strategy to connect various nanoparticle properties to nanoparticle-induced ecotoxicity. Environ. Sci. Technol..

[164] Li D., Suh S. (2019). Health risks of chemicals in consumer products: A review. Environ. Int..

[165] McGrath T.J., Ball A.S., Clarke B.O. (2017). Critical review of soil contamination by polybrominated diphenyl ethers (PBDEs) and novel brominated flame retardants (NBFRs); concentrations, sources and congener profiles. Environ. Pollut..

[166] Ji F., Wei J., Luan H., Li M., Cai Z. (2019). Study of metabolic disorders associated with BDE-47 exposure in Drosophila model by MS-based metabolomics. Ecotoxicol. Environ. Saf..

[167] Ji F., Sreenivasmurthy S.G., Wei J., Shao X., Luan H., Zhu L., Song J., Liu L., Li M., Cai Z. (2019). Study of BDE-47 induced Parkinson’s disease-like metabolic changes in C57BL/6 mice by integrated metabolomic, lipidomic and proteomic analysis. J. Hazard. Mater..

[168] Jurek A., Leitner E. (2017). Analytical determination of bisphenol A (BPA) and bisphenol analogues in paper products by GC-MS/MS. Food Addit. Contam. Part A.

[169] Vilarinho F., Sendón R., van der Kellen A., Vaz M., Silva A.S. (2019). Bisphenol A in food as a result of its migration from food packaging. Trends Food Sci. Technol..

[170] Lee S.-W., Chatterjee N., Im J.-E., Yoon D., Kim S., Choi J. (2018). Integrated approach of eco-epigenetics and eco-metabolomics on the stress response of bisphenol-A exposure in the aquatic midge Chironomus riparius. Ecotoxicol. Environ. Saf..

[171] Sheikholeslami M.N., Gómez-Canela C., Barron L.P., Barata C., Vosough M., Tauler R. (2020). Untargeted metabolomics changes on Gammarus pulex induced by propranolol, triclosan, and nimesulide pharmaceutical drugs. Chemosphere.

[172] Bonnefille B., Gomez E., Alali M., Rosain D., Fenet H., Courant F. (2018). Metabolomics assessment of the effects of diclofenac exposure on Mytilus galloprovincialis: Potential effects on osmoregulation and reproduction. Sci. Total Environ..

[173] Serra-Compte A., Álvarez-Muñoz D., Solé M., Cáceres N., Barceló D., Rodríguez-Mozaz S. (2019). Comprehensive study of sulfamethoxazole effects in marine mussels: Bioconcentration, enzymatic activities and metabolomics. Environ. Res..

[174] Hua Q., Adamovsky O., Vespalcova H., Boyda J., Schmidt J.T., Kozuch M., Craft S.L., Ginn P.E., Smatana S., Budinska E. (2021). Microbiome analysis and predicted relative metabolomic turnover suggest bacterial heme and selenium metabolism are altered in the gastrointestinal system of zebrafish (Danio rerio) exposed to the organochlorine dieldrin. Environ. Pollut..

[175] Jia M., Wang Y., Teng M., Wang D., Yan J., Miao J., Zhou Z., Zhu W. (2018). Toxicity and metabolomics study of isocarbophos in adult zebrafish (Danio rerio). Ecotoxicol. Environ. Saf..

[176] Zhang H., Aspinall J.V., Lv W., Zheng X., Zhang H., Li S., Zhang J., Bai N., Zhang Y., Wang X. (2021). Differences in kinetic metabolomics in Eisenia fetida under single and dual exposure of imidacloprid and dinotefuran at environmentally relevant concentrations. J. Hazard. Mater..

[177] Yin J., Hong X., Ma L., Liu R., Bu Y. (2020). Non-targeted metabolomic profiling of atrazine in Caenorhabditis elegans using UHPLC-QE Orbitrap/MS. Ecotoxicol. Environ. Saf..

[178] Pang M., Wang Y., Tang Y., Dai J., Tong J., Jin G. (2021). Transcriptome sequencing and metabolite analysis reveal the toxic effects of nanoplastics on tilapia after exposure to polystyrene. Environ. Pollut..

[179] Zhao Y., Qiao R., Zhang S., Wang G. (2021). Metabolomic profiling reveals the intestinal toxicity of different length of microplastic fibers on zebrafish (Danio rerio). J. Hazard. Mater..

[180] Xiang Q.-Q., Yan H., Luo X.-W., Kang Y.-H., Hu J.-M., Chen L.-Q. (2021). Integration of transcriptomics and metabolomics reveals damage and recovery mechanisms of fish gills in response to nanosilver exposure. Aquat. Toxicol..

[181] Liu W., Majumdar S., Li W., Keller A.A., Slaveykova V.I. (2020). Metabolomics for early detection of stress in freshwater alga Poterioochromonas malhamensis exposed to silver nanoparticles. Sci. Rep..

[182] Zhu Y., Wu X., Liu Y., Zhang J., Lin D. (2020). Integration of transcriptomics and metabolomics reveals the responses of earthworms to the long-term exposure of TiO2 nanoparticles in soil. Sci. Total Environ..

[183] Maria V.L., Licha D., Scott-Fordsmand J.J., Huber C.G., Amorim M.J. (2021). Multiomics assessment in Enchytraeus crypticus exposed to Ag nanomaterials (Ag NM300K) and ions (AgNO_3_)–Metabolomics, proteomics (& transcriptomics). Environ. Pollut..

[184] Liang R., Chen J., Shi Y., Lu Y., Sarvajayakesavalu S., Xu X., Zheng X., Khan K., Su C. (2018). Toxicological effects on earthworms (Eisenia fetida) exposed to sub-lethal concentrations of BDE-47 and BDE-209 from a metabolic point. Environ. Pollut..

[185] Ortiz-Villanueva E., Navarro-Martín L., Jaumot J., Benavente F., Sanz-Nebot V., Piña B., Tauler R. (2017). Metabolic disruption of zebrafish (Danio rerio) embryos by bisphenol A. An integrated metabolomic and transcriptomic approach. Environ. Pollut..

[186] Mao L., Fang S., Zhao M., Liu W., Jin H. (2021). Effects of Bisphenol A and Bisphenol S Exposure at Low Doses on the Metabolome of Adolescent Male Sprague–Dawley Rats. Chem. Res. Toxicol..

[187] Gu Y.-Y., Wei Q., Wang L.-Y., Zhang Z.-M., Zhang X.-Q., Sun A.-L., Chen J., Shi X.-Z. (2021). A comprehensive study of the effects of phthalates on marine mussels: Bioconcentration, enzymatic activities and metabolomics. Mar. Pollut. Bull..

[188] Morrison N., Bearden D., Bundy J.G., Collette T., Currie F., Davey M.P., Haigh N.S., Hancock D., Jones O.A., Rochfort S. (2007). Standard reporting requirements for biological samples in metabolomics experiments: Environmental context. Metabolomics.

[189] Spurgeon D.J., Jones O.A., Dorne J.-L.C., Svendsen C., Swain S., Stürzenbaum S.R. (2010). Systems toxicology approaches for understanding the joint effects of environmental chemical mixtures. Sci. Total Environ..

